# Sex differences in aetiology of intracerebral haemorrhage and associated small vessel disease patterns

**DOI:** 10.1093/esj/aakag069

**Published:** 2026-06-19

**Authors:** Linda Fabisch, Hatice Ozkan, Philip S Nash, Wenpeng Zhang, Martina Locatelli, Yang Du, Larysa Panteleienko, Carola Tamm, Lena Obergottsberger, Melanie Haidegger, Markus Kneihsl, Gerit Wünsch, Christian Enzinger, Robert J Simister, Hans R Jäger, Thomas Gattringer, David J Werring, Simon Fandler-Höfler

**Affiliations:** Department of Neurology, Medical University of Graz, Auenbruggerplatz 22, 8036 Graz, Austria; UCL Stroke Research Centre, Department of Translational Neuroscience and Stroke, University College London Queen Square Institute of Neurology, Queen Square, London WC1N 3BG, United Kingdom; UCL Stroke Research Centre, Department of Translational Neuroscience and Stroke, University College London Queen Square Institute of Neurology, Queen Square, London WC1N 3BG, United Kingdom; UCL Stroke Research Centre, Department of Translational Neuroscience and Stroke, University College London Queen Square Institute of Neurology, Queen Square, London WC1N 3BG, United Kingdom; UCL Stroke Research Centre, Department of Translational Neuroscience and Stroke, University College London Queen Square Institute of Neurology, Queen Square, London WC1N 3BG, United Kingdom; UCL Stroke Research Centre, Department of Translational Neuroscience and Stroke, University College London Queen Square Institute of Neurology, Queen Square, London WC1N 3BG, United Kingdom; UCL Stroke Research Centre, Department of Translational Neuroscience and Stroke, University College London Queen Square Institute of Neurology, Queen Square, London WC1N 3BG, United Kingdom; Department of Neurology, Medical University of Graz, Auenbruggerplatz 22, 8036 Graz, Austria; Department of Neurology, Medical University of Graz, Auenbruggerplatz 22, 8036 Graz, Austria; Department of Neurology, Medical University of Graz, Auenbruggerplatz 22, 8036 Graz, Austria; Department of Neurology, Medical University of Graz, Auenbruggerplatz 22, 8036 Graz, Austria; Division of Neuroradiology, Vascular and Interventional Radiology, Department of Radiology, Medical University of Graz, Auenbruggerplatz 9, 8036 Graz, Austria; Institute for Medical Informatics, Statistics and Documentation, Medical University of Graz, Auenbruggerplatz 2, 8036 Graz, Austria; Department of Neurology, Medical University of Graz, Auenbruggerplatz 22, 8036 Graz, Austria; UCL Stroke Research Centre, Department of Translational Neuroscience and Stroke, University College London Queen Square Institute of Neurology, Queen Square, London WC1N 3BG, United Kingdom; Neuroradiological Academic Unit, Department of Translational Neuroscience and Stroke, UCL Queen Square Institute of Neurology, Queen Square, London WC1N 3BG, United Kingdom; Department of Neurology, Medical University of Graz, Auenbruggerplatz 22, 8036 Graz, Austria; UCL Stroke Research Centre, Department of Translational Neuroscience and Stroke, University College London Queen Square Institute of Neurology, Queen Square, London WC1N 3BG, United Kingdom; Department of Neurology, Medical University of Graz, Auenbruggerplatz 22, 8036 Graz, Austria; UCL Stroke Research Centre, Department of Translational Neuroscience and Stroke, University College London Queen Square Institute of Neurology, Queen Square, London WC1N 3BG, United Kingdom

**Keywords:** aetiology, intracerebral haemorrhage, sex, small vessel disease

## Abstract

**Introduction:**

Causes of intracerebral haemorrhage (ICH), in particular cerebral small vessel disease (SVD), are a frequent subject of current research, yet the potential role of sex differences remains uncertain. Therefore, we aimed to investigate whether there are sex-related differences in aetiology, MRI features of SVD and risks of recurrent cerebrovascular events in patients with ICH.

**Patients and methods:**

We included patients from 2 large observational ICH study cohorts (London/UK, Graz/Austria) with available MRI. ICH aetiology was defined based on brain MRI, vascular imaging as well as clinical findings. Multivariable regression models were fitted to assess sex differences in aetiology, SVD MRI features and recurrent stroke events.

**Results:**

We identified 1043 patients (mean age 66 years, 58% male) with acute ICH. Males had a higher prevalence of cryptogenic ICH than females (aOR 1.53; 95% CI, 1.01–2.33). Males also had a higher rate of presence of any lacune (aOR 1.47; 95% CI, 1.11–1.94), severely enlarged perivascular spaces in the basal ganglia (aOR 1.45; 95% CI, 1.04–2.02) and presence of small asymptomatic diffusion-weighted imaging lesions (aOR 1.51; 95% CI, 1.06–2.15). There were no sex differences regarding recurrent ICH, incident ischaemic stroke or mortality.

**Discussion and Conclusion:**

The higher rate of presence of any lacune and enlarged perivascular spaces in men with ICH implies a higher severity of arteriolosclerosis. The mechanisms underlying the higher occurrence of cryptogenic ICH in men might include transient risk factors or incipient SVD.

## Graphical Abstract

**Graphical Abstract:**
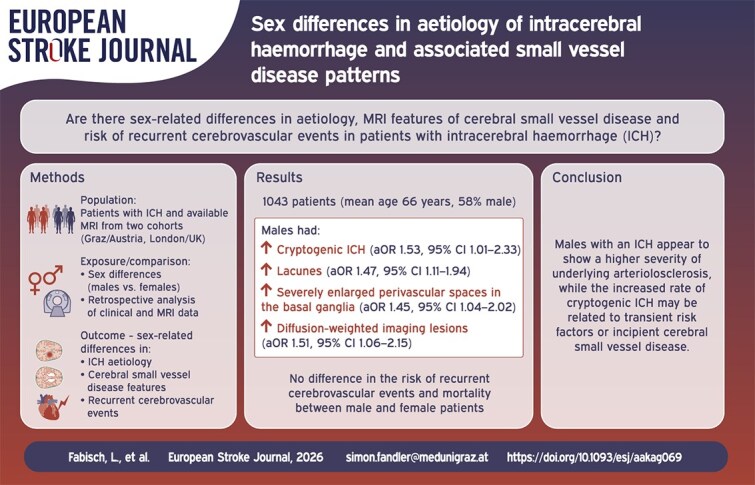

## Introduction

Intracerebral haemorrhage (ICH) is a severe stroke subtype with a global incidence of up to 28.8% among all stroke subtypes.^1^ Identifying the underlying aetiology is important since treatment, follow-up care and prognosis may vary accordingly. Cerebral small vessel disease (SVD), ie, arteriolosclerosis and cerebral amyloid angiopathy (CAA) are the main causes of nontraumatic ICH.^2^ In vivo diagnosis is currently based on imaging markers,^3^ such as a white matter hyperintensity (WMH) multispot pattern, lobar cerebral microbleeds (CMBs), enlarged perivascular spaces (ePVS) in the centrum semiovale and cortical superficial siderosis in CAA^4^ and WMH, lacunes,^5^ microbleeds mainly located in the basal ganglia, thalamus or brainstem (deep CMB) and ePVS of the basal ganglia in arteriolosclerosis.^6^ Despite thorough diagnostic work-up, the aetiology remains unknown in approximately 15%–20% of ICH patients.^7^

There is increasing interest in sex-related differences in ICH patients, since a better understanding in this regard would improve individualised risk assessment, prevention of recurrence and treatment. Previous studies found a higher age in female ICH patients^8^^,^^9^; females also tend to receive less aggressive care,^10^^,^^11^ whilst no significant sex difference was found in haematoma volume.^8^^,^^11^ Males tend to have more deep and females more lobar ICH locations,^8^^,^^9^ yet few data are currently available regarding detailed etiological sex differences—particularly concerning the prevalence of the underlying SVD patterns.

The risk of ICH recurrence is particularly high in the first year after the primary event and the risk increases with age.^12^ While previous studies mostly found no sex differences regarding the recurrence risk of ICH, higher-quality data are limited.^13^ We therefore aimed to assess potential sex differences in aetiology, SVD patterns and occurrence of vascular events in 2 large observational ICH cohorts.

## Patients and methods

### Patient selection

**Figure 1:**
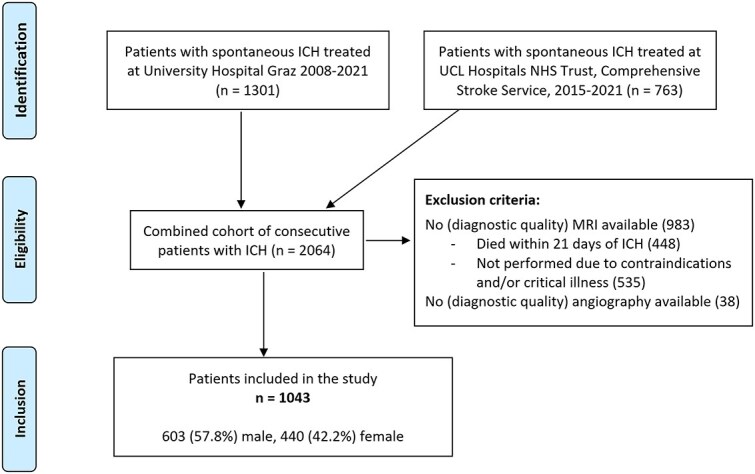
Study flowchart.

For this longitudinal multicentre study, patients with acute ICH and available MRI were included from 2 large cohorts. The Stroke Investigation Group in North and Central London (SIGNAL) registry enrolled consecutive patients with acute ICH on imaging treated at the University College London (UCL) Hospitals NHS Foundation Trust from 2015 to 2021. The Graz ICH cohort study is a database of retrospectively identified patients with first-time ICH treated at University Hospital Graz between 2008 and 2021. Screening was performed using a combination of ICD-10 diagnosis (I61.X or I62.9) and free text search in discharge letters including radiologic reports. In both cohorts, MRI was used as a clinical standard, as previously described in more detail.^14^ Patients for whom MRI was contraindicated or of insufficient quality were excluded. As we aimed to classify underlying aetiologies of ICH, we further excluded patients without any type of diagnostic quality angiography (CTA, MRA or digital subtraction angiography). A study flowchart is shown in Figure 1. Hypertension was defined as either previous diagnosis, pre-existing use of antihypertensive medication or initiation of oral antihypertensive therapy during the hospital stay that was ongoing at discharge. Diabetes was defined as ongoing or newly initiated therapy with antidiabetic drugs or haemoglobin A1c of ≥ 6.5%.

### Neuroimaging analysis

MRI protocols included at least a sequence sensitive to paramagnetic susceptibility effects (susceptibility-weighted imaging or T2^*^ gradient-echo), T2-weighted fluid attenuated inversion recovery, T2-weighted imaging and diffusion weight imaging. Haematoma location was divided into lobar, deep, cerebellar or brainstem based on the Cerebral Haemorrhage Anatomical RaTing inStrument.^15^ Aetiology was classified as CAA, arteriolosclerosis, mixed SVD, macrovascular, other secondary aetiology or cryptogenic as previously defined^16^^,^^17^: Macrovascular causes included arteriovenous malformations, dural arteriovenous fistulae, aneurysms, cavernomas and cerebral venous thromboses; other secondary causes included well-defined aetiologies distinct from those listed above and SVD (eg, tumours or vasculitis). Aetiology was classified as CAA when Boston version 2.0 criteria for probable CAA were met.^4^ In patients without lobar ICH or lobar CMB, but with presence of corresponding SVD features (at least 1 lacune, moderate or severe WMH, deep microbleeds or severely enlarged basal ganglia perivascular spaces) the cause was classified as arteriolosclerosis. Patients in whom signs of deep as well as lobar SVD were found were grouped as mixed location SVD. Patients without signs of SVD on MRI (no microbleeds, cortical superficial siderosis, lacunes, moderate/severe WMH nor severely enlarged perivascular spaces) and without a secondary cause were classified as cryptogenic.

We assessed presence and severity of markers of SVD according to the STandards for ReportIng Vascular changes on nEuroimaging criteria.^18^ The presence and distribution of CMB were rated according to the Microbleed Anatomical Rating Scale,^19^ WMH according to the Fazekas scale,^20^ and ePVS according to a validated 4-point scale.^21^ Severely ePVS were defined as > 20 PVS in 1 hemisphere. Diffusion weighed imaging (DWI) lesions were defined as increased signal intensity on DWI with corresponding decreased signal on apparent diffusion coefficient maps, remote from the index ICH lesion. MRI rating was performed blinded to clinical characteristics of patients, including sex.

Follow-up data were extracted from electronic medical records and surrounding hospital care systems, encompassing all hospitals offering acute care in the larger region. Recurrent vascular events were assessed based on medical records and confirmed by neuroimaging.

Outcome variables were the occurrence of a recurrent ICH (≥1 recurrent ICH at any time within the follow-up period), the occurrence of an ischaemic stroke (≥1 ischaemic stroke at any time within the follow-up period) and the overall mortality.

### Statistical analysis

Baseline characteristics were described using mean/SD or median/IQR for continuous variables and absolute numbers and percentages for nominal variables. Sex differences in baseline characteristics, aetiology and SVD MRI features were determined by the chi-square test and Mann–Whitney *U* test for independent samples as appropriate. Multivariable regression models were fitted for aetiology and SVD MRI features. We pre-defined a basic multivariable model (model 1) to include age and hypertension as co-variables (age being a critical confounder, hypertension the most frequent risk factor for ICH). In model 2, we additionally included co-variables with a *P*-value < .05 in univariate analysis. Where needed, we accounted for missing data using multiple imputation with chained equations (50 imputations). Outcome/target variables were not imputed. For outcome variables (recurrent ICH, ischaemic stroke and mortality), we investigated potential sex differences using multivariable Cox regression models with co-variables from model 2, as reported above. For analysis of recurrent ICH and ischaemic stroke, patients who experienced the competing risk of death were censored at the time of death.

We additionally performed a sensitivity analysis excluding patients with macrovascular and other secondary causes, repeating the same analysis steps as reported above.

IBM SPSS Statistics (Version 29) and STATA (Version 19, StataCorp LLC) were used for statistical analysis. This manuscript follows the Strengthening the Reporting of Observational Studies in Epidemiology (STROBE) reporting guideline.

## Results

We included 1043 patients with acute ICH with a mean (SD) age of 65.8 (14.6) years, 57.8% were male. Based on previously reported ICH risk factors, age, arterial hypertension, smoking, use of alcohol, drugs or any oral anticoagulants, diabetes mellitus, atrial fibrillation, heart failure, coronary artery disease, hyperlipidaemia and history of stroke/transient ischaemic attack were assessed. Arterial hypertension was the most common risk factor, followed by diabetes and smoking. Males were younger and had a higher prevalence of diabetes mellitus and coronary artery disease, heart failure, alcohol abuse and smoking. No difference was seen in the prevalence of hypertension (Table 1). MRI was performed at a median of 3 days after ICH onset (IQR 1–8), with no sex differences in timing (*P* = .27).

**Table 1:** Clinical characteristics of the study cohort according to sex.

	Total	Male	Female	*P*-value (univariate)	OR (males)	CI (95%)
***n* (%)**	1043	603 (57.8)	440 (42.2)			
**Clinical characteristics**						
**Age, y, mean (SD)**	65.8 (14.6)	64.3 (14.5)	67.8 (14.7)	<.001		
**Hypertension, *n* (%)**	761 (73.0)	450 (74.6)	311 (70.7)	.16	1.12	0.96–1.31
**Diabetes, *n* (%)**	180 (17.3)	121 (20.1)	59 (13.4)	.005	1.35	1.08–1.68
Anticoagulant use prior to ICH, *n* (%)^*^	114 (13.6)	66 (13.3)	48 (14.0)	.8	0.97	0.77–1.22
**Smoking, *n* (%)^†^**	134 (14.8)	98 (18.7)	36 (9.4)	<.001	1.67	1.25–2.23
**Alcohol abuse, *n* (%)^‡^**	90 (9.5)	77 (14.0)	13 (3.3)	<.001	3.11	1.87–5.17
**Drug abuse, *n* (%)^‡^**	17 (1.8)	11 (2.0)	6 (1.5)	.57	1.2	0.63–2.28
**Atrial fibrillation *n* (%)**	127 (12.2)	74 (12.3)	53 (12.0)	.91	1.01	0.81–1.26
**Heart failure**	24 (2.3)	19 (3.2)	5 (1.1)	.03	2.05	0.94–4.48
**Coronary artery disease^§^**	80 (8.3)	60 (10.8)	20 (5.0)	.001	1.75	1.19–2.57
**Hyperlipidaemia^§^**	120 (12.5)	74 (13.3)	46 (11.4)	.36	1.12	0.88–1.42
**History of stroke**	141 (13.5)	88 (14.6)	53 (12.0)	.24	1.14	0.91–1.43

Abbreviation: OR = odds ratio.

Missing in *n* = ^*^204 ^†^138 ^‡^94 ^§^83 patients.

### ICH location

Lobar ICH was present most frequently (44.2%), followed by deep (43.5%), cerebellar (7.4%) and brainstem ICH (4.9%). In univariate analysis, deep ICH was observed more often in males (OR 1.20; 95% CI, 1.04–1.39; *P* = .01), whilst females had higher prevalence of lobar ICH (OR 1.11; 95% CI, 1.00–1.24; *P* = .05). After adjusting for co-variables, the sex difference in deep ICH did not remain statistically significant (aOR 1.22 for men; 95% CI, 0.94–1.60; *P* = .14; Table 2).

**Table 2:** ICH location, aetiology and small vessel disease markers according to sex.

	Total	Male	Female	OR	95% CI	*P*-value univariate	aOR model 1	CI (95%) model 1	*P*-value multivariate model 1	aOR model 2	CI (95%) model 2	*P*-value multivariate model 2
**ICH location, *n* (%)**												
**Deep**	454 (43.5)	282 (46.8)	172 (39.1)	1.2	1.04–1.39	.01	1.27	0.98–1.64	.07	1.22	0.94–1.60	.14
**Lobar**	461(44.2)	251 (41.6)	210 (47.7)	0.87	0.75–1.00	.05	0.84	0.66–1.09	.19	0.89	0.68–1.16	.38
**Brainstem**	51 (4.9)	25 (4.1)	26 (5.9)	0.82	0.62–1.08	.19	0.66	0.37–1.16	.15	0.67	0.37–1.21	.18
**Cerebellar**	77 (7.4)	45 (7.5)	32 (7.3)	1.02	0.77–1.34	.91	1.07	0.66–1.72	.78	1.01	0.62–1.65	.97
**ICH aetiology, *n* (%)**												
**Cerebral amyloid angiopathy**	187 (17.9)	99 (16.4)	88 (20.0)	0.87	0.74–1.04	.14	1	0.71–1.40	1	1.02	0.72–1.46	.9
**Mixed location small vessel disease**	385 (36.9)	217 (36.0)	168 (38.2)	0.95	0.82–1.20	.47	0.92	0.71–1.20	.53	0.95	0.73–1.25	.73
**Arteriolosclerosis**	221 (21.2)	131 (21.7)	90 (20.5)	1.05	0.88–1.25	.62	1.05	0.77–1.42	.77	0.97	0.70–1.34	.85
**Macrovascular**	78 (7.5)	46 (7.6)	32 (7.3)	1.03	0.78–1.36	.83	0.95	0.57–1.59	.84	0.93	0.54–1.59	.78
**Other secondary cause**	52 (5.0)	27 (4.5)	25 (5.7)	0.87	0.65–1.17	.38	0.78	0.45–1.38	.4	0.79	0.44–1.42	.44
**Cryptogenic**	120 (11.5)	83 (13.8)	37 (8.4)	1.42	1.07–1.87	.007	1.53	1.01–2.33	.046	1.47	0.95–2.27	.085
**Small vessel disease markers, *n* (%)**												
**Any microbleeds**	635 (60.9)	363 (60.2)	272 (61.8)	0.96	0.83–1.11	.6	1.01	0.78–1.31	.96	1.04	0.79–1.36	.79
**Any lobar microbleeds**	486 (46.6)	276 (45.8)	210 (47.7)	0.96	0.83–1.10	.53	1.02	0.79–1.32	.88	1.07	0.82–1.40	.6
**Any deep microbleeds**	411 (39.4)	227 (37.6)	184 (41.8)	0.91	0.78–1.04	.17	0.81	0.62–1.04	.1	0.87	0.67–1.13	.31
**Cortical superficial siderosis**	121 (11.6)	68 (11.3)	53 (12.0)	0.96	0.77–1.19	.7	1.18	0.79–1.76	.42	1.25	0.83–1.88	.28
**Disseminated cortical superficial siderosis**	67 (6.4)	38 (6.3)	29 (6.6)	0.97	0.73–1.29	.84	1.24	0.74–2.08	.42	1.37	0.81–2.32	.24
**Moderate-to-severe white matter hyperintensities (Fazekas 2–3)**	538 (51.6)	301 (49.9)	237 (53.9)	0.91	0.79–1.05	.21	0.98	0.76–1.28	.89	1.01	0.77–1.32	.97
**Any lacune**	320 (30.7)	202 (33.5)	118 (26.8)	1.21	1.02–1.42	.02	1.47	1.11–1.94	.006	1.42	1.06–1.89	.02
Enlarged perivascular spaces in the centrum semiovale^*^	336 (36.4)	186 (35.3)	150 (37.9)	0.94	0.81–1.09	.42	1.01	0.77–1.34	.93	1.07	0.80–1.43	.65
**Enlarged perivascular spaces in the basal ganglia^†^**	218 (23.1)	130 (24.0)	88 (21.8)	1.08	0.90–1.29	.43	1.45	1.04–2.02	.03	1.47	1.05–2.07	.03
**Diffusion-weighted imaging lesion^‡^**	164 (16.1)	109 (18.4)	55 (12.9)	1.3	1.03–1.63	.02	1.51	1.06–2.15	.02	1.46	1.01–2.10	.04

Abbreviations: aOR = adjusted odds ratio; OR = odds ratio.

Missing in *120 †99 ‡22 patients.

Model 1: adjusted for age and arterial hypertension.

Model 2: adjusted for age, arterial hypertension, diabetes, alcohol, smoking, heart failure and coronary artery disease.

### Sex differences in aetiology

Mixed location SVD was the most common aetiological subgroup in both sexes (36.0% in males; 38.2% in females), followed by arteriolosclerosis and CAA.

There were no significant differences in the prevalence of SVD-related aetiologies of ICH between males and females. In univariate analysis, we observed a higher occurrence of cryptogenic ICH in males (OR 1.42; 95% CI, 1.07–1.87; *P* = .007). These results remained statistically significant in multivariable analysis adjusted for age and hypertension (aOR 1.53; 95% CI, 1.01–2.33; *P* = .04), but were not significant in multivariable analysis adjusted for other co-morbidities (aOR1.47; 95% CI, 0.95–2.27; *P* = .085; Table 2).

### Sex differences in small vessel disease markers

The most common SVD marker in both sexes was microbleeds (60.9%), followed by severe WMH (51.6%) and severely enlarged perivascular spaces in the centrum semiovale (36.4%).

After adjusting for co-variables, males had a higher rate of presence of any lacune (aOR = 1.42; 95% CI, 1.06–1.89; *P* = .02), severely enlarged perivascular spaces in the basal ganglia (aOR = 1.47; 95% CI, 1.05–2.07; *P* = .03) and presence of small asymptomatic DWI lesions (aOR = 1.46; 95% CI, 1.01–2.10; *P* = .04). No differences were found regarding microbleeds, cortical superficial siderosis or white matter hyperintensities (Table 2).

### Follow-up

**Figure 2:**
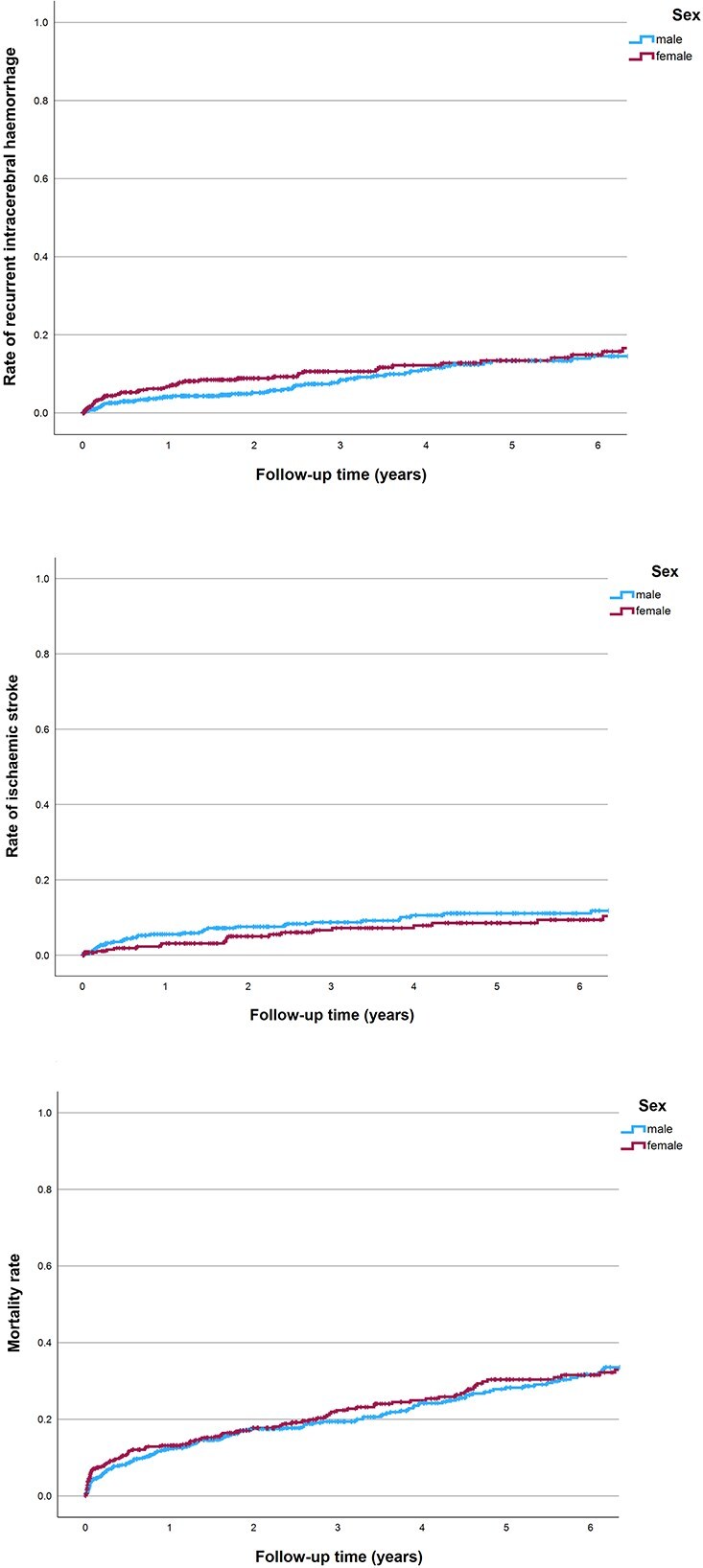
Recurrent ICH, ischaemic stroke and mortality during the follow-up period.

All 1043 patients had available follow-up data. The median follow-up time was 2.2 years (IQR 0.4–5.7). In the follow-up period, 102 recurrent ICH and 62 ischaemic strokes occurred, 253 patients died. No sex difference was found regarding ICH recurrence, occurrence of ischaemic stroke and mortality during the follow-up period after correction for age and comorbidities as above (Table 3; Figure 2).

**Table 3:** Cox regression analysis with sex as target variable.

*n* = 1043	Adjusted hazard ratios for women (95% CI)	*P*-value
**Recurrence of ICH**	1.20 (0.78–1.87)	.41
**Ischaemic stroke**	0.81 (0.46–1.41)	.45
**Mortality**	0.94 (0.70–1.26)	.66

Adjusted for age, arterial hypertension, diabetes, alcohol, smoking, heart failure and coronary artery disease.

### Sensitivity analysis

In a sensitivity analysis excluding patients with macrovascular and other secondary causes, differences in clinical characteristics between males and females were unchanged (Table S1). Similar to the full study population, we found that males had higher rates of presence of any lacune and severely enlarged perivascular spaces in the basal ganglia (Table S2). In addition, we found a slightly lower rate of any deep microbleeds and a lower rate of brainstem ICH in men.

## Discussion

In a large 2-centre study of patients with ICH, we demonstrated sex differences in aetiology according to SVD MRI features in a large multicentre cohort of ICH patients. First, we found a higher occurrence of cryptogenic ICH in males. Second, certain markers of arteriolosclerosis (lacunes and enlarged perivascular spaces in the basal ganglia) were more frequently found in men.

A possible explanation for the increased occurrence of cryptogenic ICH in males is a higher burden of incipient arteriolosclerosis—not yet apparent on conventional brain MRI, due to a higher percentage of SVD risk factors in males possibly reinforced by a lower adherence to risk management in males, as previously shown in patients with arterial hypertension.^22^ However, transient or unrecognised risk factors (eg, the use of recreational drugs such as cocaine, amphetamines or other sympathomimetic agents, which are more frequent in men^23^^,^^24^) might also have played a role. For example, while we adjusted for alcohol and recreational drug use, which are more frequent in men,^25^ it might have been preferentially unrecognised or denied by men since women seem to report recreational drug use more accurately.^26^ A case-crossover study identified several potential transient ICH trigger factors, most of which (eg, caffeine consumption, sexual activity) are known to be associated with temporarily elevated blood pressure. Although the study did not differentiate between females and males, some included risk factors, such as heavy lifting, could possibly apply more frequently to males; however, no clear data on this is available.^27^

Men exhibited a higher prevalence of diabetes mellitus, coronary heart disease and heart failure, which may partly be explained by more frequent smoking and alcohol consumption and may also contribute to the higher burden of arteriolosclerosis-related SVD features. Diabetes mellitus has been shown to induce endothelial dysfunction,^28^ thereby contributing to the development and progression of arteriolosclerosis. Lacunes and enlarged perivascular spaces in the basal ganglia, both markers of arteriolosclerosis, were found more frequently in males. A higher burden of lacunes in male patients was also found in a large pooled patient analysis of patients with ischaemic stroke.^29^ Regarding ePVS, a pooled analysis of multiple cohort studies showed more ePVS in the mesencephalon, centrum semiovale and basal ganglia in males,^30^ while in another study of CAA patients, ePVS in the centrum semiovale were found more often in females.^31^ While more exposure to risk factors or genetic susceptibility might be a potential explanation, it is important to mention that other markers of arteriolosclerosis/SVD (such as CMB or WMH) were not different between the sexes in our study.

Previous studies found females to have a higher progression rate and degree of WMH^32^^,^^33^ and to be more frequently affected by moderate-to-severe WMH than males.^29^ Regarding CMB, available data in literature are divergent: A cohort study including over 20,000 patients with ischaemic stroke found CMB to be more frequent in males,^29^ while population-based studies demonstrated a similar degree of CMB at baseline.^34^ Variation in results is likely to be explained by different study settings (population-based vs ischaemic stroke vs ICH cohorts). However, most studies show a similar pattern of more lacunes and/or CMB in men, and more severe WMH in women, indicating sex differences in SVD pathophysiology.

We found higher occurrence of deep ICH in males and lobar ICH in females in univariate analysis, although no differences remained statistically significant after adjusting for age. This is likely explained by the higher proportion of CAA-associated lobar ICH in elderly patients. Previous studies frequently showed differences according to sex similar to our results,^8^^,^^35^^,^^36^ although sex differences in ICH location may differ depending on the population studied.^37^ One possible explanation for these findings is a higher prevalence of hypertension, a major risk factor for arteriolosclerosis-associated deep ICH, in males on a population level,^38^ although in our ICH cohort we did not find a difference in the prevalence of hypertension according to sex. Despite more lobar ICH and older age in females, there was no significant sex difference in frequency of CAA, which is consistent with previously reported data.^8^

We found no significant sex difference in the recurrence of cerebrovascular events during follow-up. Similarly, a study including data from the Swedish National Stroke Registry found no significant sex differences in functional outcome and survival at 3-month follow-up.^39^ In contrast, a meta-analysis including 32 studies showed a better functional outcome in males at 3-month follow-up.^13^ Female ICH patients tend to be older,^8^ less likely to be treated in a stroke unit^39^ and more likely to receive early do-not-resuscitate orders,^10^^,^^11^ which might have contributed to those results. As our study only included patients with ICH who had MRI, those with most severe haemorrhages (and highest risk for in-hospital mortality) were largely not assessed.

The main strength of our study is the large cohort of 1043 patients with detailed clinical and neuroradiological (MRI) data collection over a decade in a multicentre cohort. Our study has some limitations, including inconsistencies in follow-up time, variation in MRI protocols over time (including scanner field strength and type of blood-sensitive sequence used) and possible variations in the degree of aetiological work-up between patients, which might affect the diagnosis of specific ICH causes (and cryptogenic ICH). Limiting this study to patients with available MRI introduces a bias towards milder manifestations of ICH. We had no data regarding functional outcome at follow-up, which might have been of interest. Due to the explorative nature of our study, we chose not to correct for multiple comparisons, which implies chance findings are possible. Therefore, findings reaching nominal significance (*P* < .05) should be interpreted as hypothesis-generating and require validation in independent cohorts. Furthermore, SVD markers were operationalised as binary variables, without incorporating differences in severity, which may have led to a reduction in analytical sensitivity. Although our analyses adjusted for relevant covariates, residual confounding may still have affected our findings.

## Conclusion

In conclusion, while the distribution of ICH aetiologies and SVD markers were largely similar between males and females, we found some sex differences in aetiology and SVD phenotypes indicating higher burden of severe arteriolosclerosis and cryptogenic ICH in men. Further research, including longitudinal studies investigating SVD progression is necessary to improve the understanding of sex differences in underlying pathological processes leading to ICH, and in order to optimise risk assessment, primary and secondary prevention.

## Supplementary Material

Supplementary_Material_aakag069

## Data Availability

Datasets generated during this study are available from the corresponding author upon reasonable request.
