# Supplementary material for: Sex differences in aetiology of intracerebral haemorrhage and associated small vessel disease patterns
Source: Eur Stroke J. 2026 Jun 19;11(6):aakag069. doi: 10.1093/esj/aakag069 (PMC13280940; doi:10.1093/esj/aakag069)
Supplement: Supplementary_Material_aakag069 [file supplementary_material_aakag069.docx]

**Sex differences in aetiology of intracerebral haemorrhage and associated small vessel disease patterns**

**Supplementary material:**

- Supplementary table 1: Clinical characteristics according to sex in patients without macrovascular or other secondary cause
- Supplementary table 2: ICH location, aetiology and small vessel disease markers according to sex in patients without macrovascular or other secondary cause

| **Supplementary table 1:** Clinical characteristics according to sex in patients without macrovascular or other secondary cause | | | | | | |
| --- | --- | --- | --- | --- | --- | --- |
|  | Total | Male | Female | p-Value  Univariate | OR (Males) | CI (95%) |
|  |  |  |  |  |  |  |
| **Clinical characteristics** | | | | | | |
| Age, y, mean (SD) | 67.2 (13.6) | 65.2 (13.9) | 69.9 (12.8) | <0.001 |  |  |
| Hypertension, n (%) | 699 (76.6) | 415 (78.3) | 284 (74.2) | 0.14 | 1.14 | 0.96-1.35 |
| Diabetes, n (%) | 165 (18.1) | 109 (20.6) | 56 (15.6) | 0.02 | 1.29 | 1.03-1.62 |
| Anticoagulant use prior to ICH, n (%) * | 99 (13.6) | 53 (12.2.) | 46 (15.6) | 0.19 | 0.85 | 0.67-1.07 |
| Smoking, n (%)† | 111 (14.1) | 81 (17.7) | 30 (9.1) | <0.001 | 1.6 | 1.19-2.25 |
| Alcohol abuse, n (%)‡ | 82 (9.9) | 70 (14.5) | 12 (3.5) | <0.001 | 3.05 | 1.80-5.18 |
| Drug abuse, n (%) ‡ | 13 (1.6) | 7 (1.4) | 6 (1.7) | 0.74 | 0.90 | 0.50-1.63 |
| Atrial fibrillation, n (%) | 119 (13.0) | 70 (13.2.) | 49 (12.8) | 0.86 | 1.02 | 0.81-1.29 |
| Heart failure, n (%) | 22 (2.4) | 18 (3.4) | 4 (1.0) | 0.02 | 2.34 | 0.96-5.70 |
| Coronary artery disease, n (%)§ | 70 (8.4) | 53 (10.9) | 17 (4.9) | 0.002 | 1.78 | 1.17-2.72 |
| Hyperlipidaemia, n (%)§ | 113 (13.5) | 68 (14.0) | 45 (12.9) | 0.65 | 1.06 | 0.83-1.35 |
| History of stroke, n (%) | 133 (14.6) | 80 (15.1) | 53 (13.8) | 0.60 | 1.06 | 0.85-1.33 |
| Missing in n= *185 †126 ‡83 §78 patients | | | | | | |

| **Supplementary table 2:** ICH location, aetiology and small vessel disease markers according to sex in patients without macrovascular or other secondary cause | | | | | | | | | |
| --- | --- | --- | --- | --- | --- | --- | --- | --- | --- |
|  | Total | Male | Female | p-Value  Univariate | OR (Males) | 95% CI | p-Value  Multivariate Model 1 | OR (Males)  Model 1 | CI (95%)  Model 1 |
| **ICH location, n (%)** |  |  |  |  |  |  |  |  |  |
| Deep | 433 (47.4) | 270 (50.9) | 163 (42.6) | 0.01 | 1.22 | 1.04-1.42 | 0.14 | 1.23 | 0.93-1.61 |
| Lobar | 382 (41.8) | 209 (39.4) | 173 (45.2) | 0.08 | 0.87 | 0.75-1.02 | 0.44 | 0.90 | 0.68-1.18 |
| Brainstem | 33 (3.6) | 13 (2.5) | 20 (5.2) | 0.03 | 0.68 | 0.51-0.91 | 0.03 | 0.45 | 0.22-0.93 |
| Cerebellar | 65 (7.1) | 38 (7.2) | 27 (7.0) | 0.94 | 1.01 | 0.75-1.36 | 0.78 | 1.08 | 0.64-1.81 |
| **ICH aetiology, n (%)** |  |  |  |  |  |  |  |  |  |
| Cerebral amyloid angiopathy | 187 (20.5) | 99 (18.7) | 88 (23.0) | 0.11 | 0.86 | 0.72-1.03 | 0.93 | 1.02 | 0.72-1.44 |
| Mixed location small vessel disease | 385 (42.2) | 217 (40.9) | 168 (43.9) | 0.38 | 0.93 | 0.80-1.09 | 0.38 | 0.89 | 0.67-1.16 |
| Arteriolosclerosis | 221 (24.2) | 131 (24.7) | 90 (23.5) | 0.67 | 1.04 | 0.87-1.25 | 0.96 | 0.99 | 0.72-1.36 |
| Cryptogenic | 120 (13.1) | 83 (15.7) | 37 (9.7) | 0.008 | 1.42 | 1.07-1.87 | 0.18 | 1.35 | 0.87-2.08 |
| **Small vessel disease markers, n (%)** |  |  |  |  |  |  |  |  |  |
| Any microbleeds | 604 (66.2) | 343 (64.7) | 261 (68.1) | 0.28 | 0.91 | 0.77-1.08 | 0.60 | 0.93 | 0.70-1.23 |
| Any lobar microbleeds | 460 (50.4) | 259 (48.9) | 201 (52.5) | 0.28 | 0.92 | 0.79-1.07 | 0.87 | 0.98 | 0.74-1.28 |
| Any deep microbleeds | 394 (43.2) | 216 (40.8) | 178 (46.5) | 0.09 | 0.87 | 0.75-1.02 | 0.02 | 0.73 | 0.55-0.96 |
| Cortical superficial siderosis | 119 (13.0) | 67 (12.6) | 52 (13.6) | 0.68 | 0.95 | 0.77-1.19 | 0.38 | 1.20 | 0.80-1.81 |
| Disseminated cortical superficial siderosis | 67 (7.3) | 38 (7.2) | 29 (7.6) | 0.81 | 0.97 | 0.73-1.28 | 0.40 | 1.25 | 0.74-2.11 |
| Moderate-to-severe white matter hyperintensities (Fazekas 2-3) | 519 (56.8) | 289 (54.5) | 230 (60.1) | 0.10 | 0.88 | 0.75-1.03 | 0.71 | 0.95 | 0.72-1.25 |
| Any lacune | 307 (33.6) | 192 (36.2) | 115 (30.0) | 0.05 | 1.18 | 1.00-1-40 | 0.02 | 1.42 | 1.06-1.89 |
| Enlarged perivascular spaces in the centrum semiovale * | 311 (38.8) | 168 (36.6) | 143 (41.8) | 0.13 | 0.88 | 0.75-1.04 | 0.61 | 0.93 | 0.69-1.25 |
| Enlarged perivascular spaces in the basal ganglia † | 209 (25.4) | 125 (26.4) | 84 (24.1) | 0.44 | 1.08 | 0.89-1.30 | 0.02 | 1.49 | 1.06-2.09 |
| Diffusion-weighted imaging lesion ‡ | 148 (16.6) | 97 (18.7) | 51 (13.7) | 0.05 | 1.25 | 0.99-1.58 | 0.10 | 1.37 | 0.94-1.99 |
| Missing in *112 †91 ‡22 patients | | | | | | | | | |
